# Bacillus thuringiensis Bacteremia in a 30-Year-Old Intravenous Drug User: A Report of a Rare Case

**DOI:** 10.7759/cureus.71704

**Published:** 2024-10-17

**Authors:** Salim Barakat, Hyunwoo Kim, Razan Dankar, Chadik Hewlett

**Affiliations:** 1 Internal Medicine, Northwell Health/Staten Island University Hospital, Staten Island, USA; 2 Internal Medicine, Touro College of Osteopathic Medicine, New York, USA

**Keywords:** bacillus thuringiensis, bacteremia, gram positive bacteria, iv drug abusers, microbial contamination

## Abstract

*Bacillus thuringiensis* is a gram-positive bacterium used in agriculture, with rare human infections that typically occur in immunocompromised individuals through environmental exposure. This report discusses the case of a 30-year-old intravenous drug user and polysubstance abuser who developed bacteremia due to *Bacillus thuringiensis*. The patient originally presented with fever and leg ulcers from injection sites, and he was successfully treated with vancomycin. This case underscores the importance of recognizing atypical pathogens like *Bacillus thuringiensis* in intravenous drug users, particularly when contaminated drug paraphernalia is involved. Although generally low in virulence, this bacterium can cause systemic infections under certain conditions. Prompt identification and treatment are crucial to prevent complications, highlighting the need for increased clinical awareness and appropriate microbiological investigations in this vulnerable population.

## Introduction

The *Bacillus cereus* group comprises several related species of gram-positive, spore-producing bacteria. *Bacillus cereus, Bacillus anthracis, and Bacillus thuringiensis* (Bt) are the most extensively studied bacteria among this group. *Bacillus anthracis *and* Bacillus cereus* are more commonly related to human infections, whereas Bt is mostly used in pest and insect control [1,2]. The latter is characterized by its ability to produce insecticidal proteins and toxins. Though closely related to *Bacillus cereus*, which is associated with food poisoning, Bt rarely causes human infections [1]. Nevertheless, there have been isolated reported cases of Bt bacteremia, particularly in immunocompromised or neutropenic individuals. Such cases have been usually associated with environmental exposure [3-5].

Similar to immunodeficiency, intravenous drug use also significantly increases the risk of both common and rare infections. This usually occurs due to unsterile injection practices, contaminated needles, or compromised immunity. A notable case by Kalinoski in 2023 involved fatal Bt bacteremia in an intravenous drug user (IVDU) [6]. Here, we report another rare case of Bt bacteremia in a 30-year-old IVDU with a history of schizoaffective disorder and polysubstance use disorder, illustrating the need for clinical awareness of atypical pathogens in this patient population.

## Case presentation

A 30-year-old male with a past medical history of schizoaffective disorder, positive hepatitis C antibody, polysubstance abuse, and IVDU, and a past surgical history of splenectomy presented to the emergency department (ED) with subjective complaints of fever and chills. The patient reported no other symptoms. Physical examination was significant for bilateral lower leg ulcerated wounds from intravenous injections in these areas.

**Figure 1:**
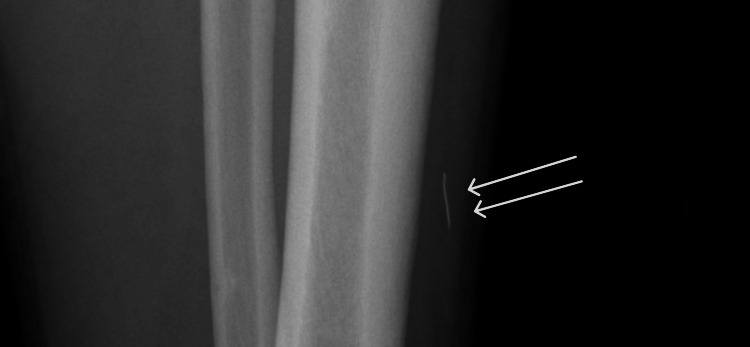
Left leg X-ray. The white arrows point toward the foreign body object.

Vital signs in the ED indicated a temperature of 101°F, blood pressure of 116/67 mmHg, heart rate of 91 beats per minute, and respiratory rate of 20 breaths per minute. Initial laboratory workup, including a complete blood count, basic metabolic panel, and liver function tests, was within normal limits. A chest X-ray showed no abnormalities. X-ray of the bilateral leg wounds revealed a linear metallic density anterior to the distal shaft of the left fibula, likely suspicious for a needle used as drug paraphernalia (Figure 1). Blood cultures were obtained due to the patient’s history of IVDU and presenting symptoms of subjective fever and chills. The patient then left the ED against medical advice 4 hours after being admitted. Within four days, blood cultures (two sets) grew *Bacillus* species, later identified as Bt through biochemical and molecular testing.

Upon identification of Bt in blood cultures, the patient was contacted and readmitted to the hospital. The patient was still complaining of fever and chills during that time due to this bacteremia. His blood test now was significant for leukocytosis with left shift and an elevated erythrocyte sedimentation rate (ESR). He was started on a seven-day course of vancomycin at a dosage of 15 milligrams per kilogram (mg/kg) every 12 hours treating Bt bacteremia.

**Figure 2:**
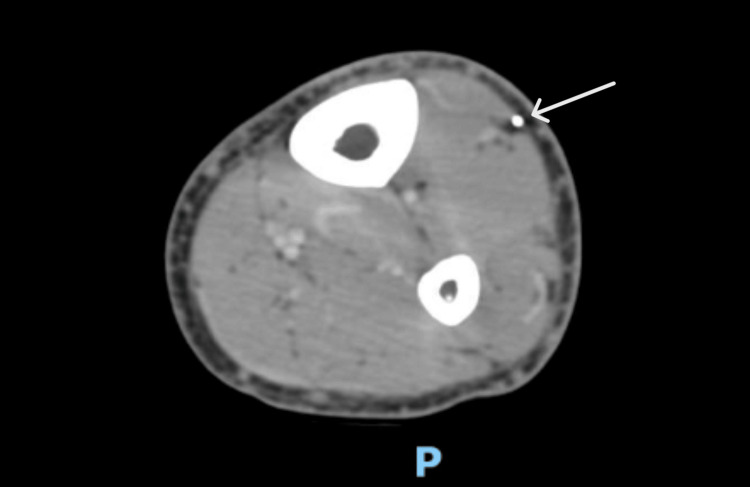
CT scan of the left lower extremity. Axial view of the image. The white arrow points toward the foreign body object. CT, computed tomography.

Computed tomography (CT) scan of the lower extremities (Figure 2) confirmed the foreign body object and ruled out osteomyelitis and deep tissue infection. Wound swabs obtained during this admission showed multiple bacteria (*Staphylococcus epidermis, Escherichia coli,* and *Propionibacterium acnes)*, suggesting contamination. Transthoracic echocardiogram showed normal biventricular function with no vegetations. The patient was monitored closely for any signs of systemic infection or complications. Surgery to remove the foreign body was offered, but the patient’s family declined, citing concerns that the skin graft might not be successful if the patient continued intravenous drug use.

The patient’s condition remained stable throughout the hospital stay, with no fever or hemodynamic instability after antibiotic initiation. Repeated blood cultures were negative after initiation of vancomycin. The patient again left against medical advice in stable condition with instructions for outpatient follow-up and continued psychiatric care for schizoaffective disorder and substance use disorder counseling.

## Discussion

IVDU poses significant risks for various infections, some of which are rare but serious. These infections occur due to unsterile injection practices, contaminated needles, or compromised immune systems [7,8]. 

One of the common infections associated with IVDU is endocarditis, a potentially life-threatening infection of the heart valves [9]. Rare infections linked to IVDU include necrotizing fasciitis which is a rapidly progressing soft-tissue infection caused by bacteria like *Streptococcus pyogenes *or *Clostridium perfringens*. Botulism, caused by *Clostridium botulinum*, can also occur when injecting contaminated substances, leading to severe muscle paralysis [10]. Fungal infections such as candidemia, an invasive infection caused by *Candida* species, and atypical bacterial infections like *Bacillus cereus* or *Mycobacterium abscessus* have also been reported in IVDUs, especially when the drug solutions or injection environments are not sterile [11].

This case highlights the rare occurrence of Bt bacteremia in the context of IVDU. Bt is usually considered a low-virulence organism, but it has been implicated with sepsis as a source of nosocomial infection in critically ill patients [3]. In our literature review, the only case of Bt bacteremia in an IVDU was reported by Kalinoski in 2023 [6]. Taking into account the eventual expiration of the patient in the mentioned case, the identification of Bt in IVDUs necessitates a focus on medical management rather than attributing it to be due to contaminated injection materials. In our patient, the likelihood of the infection being secondary to contamination should be considered, given that he had a foreign body material in his legs secondary to IVDU. However, cultures from the wound did not reveal Bt, making the case more exceptional and bizarre. Fortunately, our patient started to feel better, and his fever and chills resolved after starting intravenous vancomycin. 

The management of Bt infections remains challenging due to limited clinical data and the rarity of reported cases [12]. No guidelines exist for the treatment of invasive Bt infections. However, published susceptibility reports describe the complete susceptibility of *Bacillus cereus* to vancomycin, quinolones, gentamicin, carbapenems, and tigecycline [13]. In this patient, early recognition and broad antibiotic therapy with vancomycin led to a favorable outcome without complications.

This case provides an important learning point in increasing awareness of the more uncommon organisms causing bacteremia and even possible morbidity as in the case reported by Kalinoski [6]. As the literature on Bt bacteremia is extremely limited, especially in the IVDU population, it is important to consider Bt within the differentials rather than presumptively dismissing it as a contaminant similar to its related organism,* Bacillus cereus*. It is prudent to weigh the clinical context as both these *Bacillus* organisms can cause significant distress.

## Conclusions

Bt* *bacteremia is a rare infection that can be seen in immunocompromised patients and the IVDU population. This case report of Bt* *bacteremia* *in an IVDU highlights the need for clinical vigilance and appropriate microbiological investigation for proper management. Early identification and tailored therapy are crucial to prevent potential complications and improve outcomes in this unique patient population.
